# Impact of Age, Sex, and Renal Function on the Efficacy and Safety of Direct Oral Anticoagulants vs. Vitamin K Antagonists for the Treatment of Acute Venous Thromboembolism: A Meta-Analysis of 22,040 Patients

**DOI:** 10.3389/fcvm.2021.700740

**Published:** 2021-09-08

**Authors:** Bo Zhou, Haoyu Wu, Chen Wang, Bowen Lou, Jianqing She

**Affiliations:** ^1^Respiratory and Critical Care Medicine, The First Affiliated Hospital of Xi'an Jiaotong University, Xi'an, China; ^2^Cardiovascular Department, The First Affiliated Hospital of Xi'an Jiaotong University, Xi'an, China; ^3^Key Laboratory of Environment and Genes Related to Diseases, Ministry of Education, Xi'an, China

**Keywords:** venous thromboembolism, direct oral anticoagulants, vitamin K antagonists, meta-analysis, efficacy, safety

## Abstract

**Objective:** In this study, we conducted a meta-analysis to assess the impact of age, sex, and renal function on the efficacy and safety of direct oral anticoagulants (DOACs) vs. vitamin K antagonists (VKAs) for the treatment of acute venous thromboembolism (VTE).

**Methods:** Electronic databases (accessed till June 2021) were systematically searched to investigate randomized clinical trials evaluating apixaban, dabigatran, edoxaban, and rivaroxaban vs. VKAs for the treatment of acute VTE. Results were presented as odds ratio (OR) and 95% CIs.

**Results:** Direct oral anticoagulants were associated with a borderline higher efficacy in women (OR: 0.79, 95% CI: 0.62–1.02), a significantly higher efficacy in patients with age more than 75 years (OR: 0.51, 95% CI: 0.32–0.80), and creatinine clearance <50 ml/min (OR: 0.57, 95% CI: 0.32–0.99). The primary safety endpoint of major or clinically relevant non-major bleeding was significantly reduced in DOACs as compared to VKAs in both patients with age <75 years (OR: 0.79, 95% CI: 0.70–0.89) and patients with age more than 75 years (OR: 0.75, 95% CI: 0.59–0.96). DOACs also show an advantage in terms of major or clinically relevant non-major bleeding in men (OR: 0.72, 95% CI: 0.60–0.86) and patients with creatinine clearance of more than 50 ml/min (OR: 0.75, 95% CI: 0.67–0.84).

**Conclusions:** Direct oral anticoagulants have exhibited clinical preference among patients with acute VTE with decreased thrombosis and bleeding events, especially in patients with age more than 75 years and creatinine clearance <50 ml/min.

## Introduction

Venous thromboembolism (VTE) refers to a blood clot in the vein. It is defined by deep vein thrombosis (DVT) and pulmonary embolism (PE), which is the third leading vascular disease after heart attack and stroke. The most common triggers for VTE are surgery, cancer, immobilization, and hospitalization. The patients with VTE exhibit a high risk of recurrence after the first event. Recent studies showed that about 10% of patients with VTE had recurrence within a year (1, 2). Moreover, VTE is associated with long-term, clinically significant complications, namely, chronic persistent thromboembolic pulmonary hypertension or post-thrombotic syndrome. Therefore, VTE has laid a substantial personal and economic burden and is associated with a high risk of mortality in patients.

Vitamin K antagonists (VKAs) have been considered as an effective anticoagulation treatment of VTE for many years. However, VKA treatment has two limitations, the rate of major bleeding complications (2.1%) during the first 6 months and frequently monitoring international normalized ratio (INR) (3), for which physicians have to utilize it with caution. The treatment of VTE depends on a balance between the prevention of recurrence and the incidence of bleeding complications (4). In recent years, direct oral anticoagulants (DOACs) have been thought as a preferable treatment to VTE due to their similar efficacy and lower risks of bleeding complications as compared to warfarin and no need to monitor INR (5). However, detailed knowledge about the safety and efficacy of DOACs as compared to VKA remains limited in patients with acute VTE especially in the elderly and in patients with decreased renal functions.

So far, four randomized clinical trials (RCTs) have evaluated the utilization of DOACs in patients with acute VTE, the AMPLIFY (apixaban) (6), the EINSTEIN (rivaroxaban) (7), the HOKUSAI (edoxaban) (8), and RE-COVER (dabigatran) (9). Subgroup analysis of the above studies has also been released concerning different age groups, sex, and renal function. However, because of the limited sample size of the investigated subgroups, the superiority or non-inferiority results may not be statistically powered. Thus, a meta-analysis of the subgroups in four studies might provide more information as to the safety and efficacy of DOAC in patients with VTE.

Moreover, in real-world practice, many physicians do not prefer to prescribe DOACs in patients with advanced age, reduced renal function, or multiple comorbidities. In this scenario, VKA is preferred concerning the limited data on the safety and efficacy of DOAC in the elderly and patients with reduced renal function. However, these patients are known to be at higher risk for both anticoagulation-related bleeding and VTE recurrence, who might benefit more from DOAC and thus warrants more clinical evidence. Therefore, in this study, we conducted a meta-analysis to assess the impact of age, sex, and renal function on the efficacy and safety of DOACs vs. VKAs for the treatment of acute VTE.

## Methods

### Search Strategy and Study Selection

The search was conducted based on PubMed, Embase, and ISI Web of Science up to June 31, 2021, by two authors independently. In addition, Google Scholar was used as a supplement. To reduce publication bias, language or year was not restricted in the search. We included studies that had described the impact of age, sex, and renal function on the efficacy and safety of DOACs vs. VKAs for the treatment of acute VTE as the primary endpoint or in the subgroup analysis. Case reports and real-world analysis were excluded. The search involved the keywords: “NOAC,” “DOAC,” “oral anticoagulants,” “rivaroxaban,” “dabigatran,” “apixaban,” OR “edoxaban”; “vitamin K antagonist,” “VKA,” OR “warfarin”; and “venous thromboembolism,” “VTE,” “deep vein thrombosis,” “DVT,” “pulmonary embolism,” OR “PE”). We assessed the reference lists of the retrieved articles and relevant reviews for additional published and unpublished data. This meta-analysis was performed following the Preferred Reporting Items for Systematic Reviews and Meta-Analyses guidelines. Data extracted from included studies and data used for all analyses are publicly available.

### Outcome Measures

We extracted data on the primary efficacy and safety outcome. The primary efficacy outcome was the occurrence of symptomatic recurrent VTE (defined as DVT and/or fatal/nonfatal PE) during the study period. The principal safety outcome included the incidence of clinically relevant bleeding (defined by the composite of major and clinically relevant non-major bleeding). The endpoint of major bleeding was defined as obvious bleeding presented with a reduction of hemoglobin ≥2 g/dl or requiring transfusion of two or more units of blood, occurring at a critical site, or contributing to death. Clinically relevant non-major bleeding was described as obvious bleeding that did not meet the criteria for major bleeding but required medical intervention, clinical inspection, interruption of study drug, or impairment of daily activities.

The data were extracted by two investigators independently, who conducted the data collection and the methodological quality assessment. The data collection was under the Quality of Reporting of Meta-Analyses statement.

### Statistical Analysis

The meta-analysis was performed following the Cochrane Handbook for Systematic Reviews of Interventions. Odds ratios (ORs) were calculated with fixed-effects models, and the results were presented as pooled ORs and 95% CIs. The calculations for the meta-analysis were conducted with Review Manager (version 5.3 for Windows, Cochrane Collaboration, Oxford, United Kingdom). This study followed the Quality of Reporting of Meta-Analyses and Cochrane Collaboration guidelines for reporting meta-analyses.

### Patient and Public Involvement

There was no direct patient or public involvement in this review.

## Results

### Literature Search

**Figure 1:**
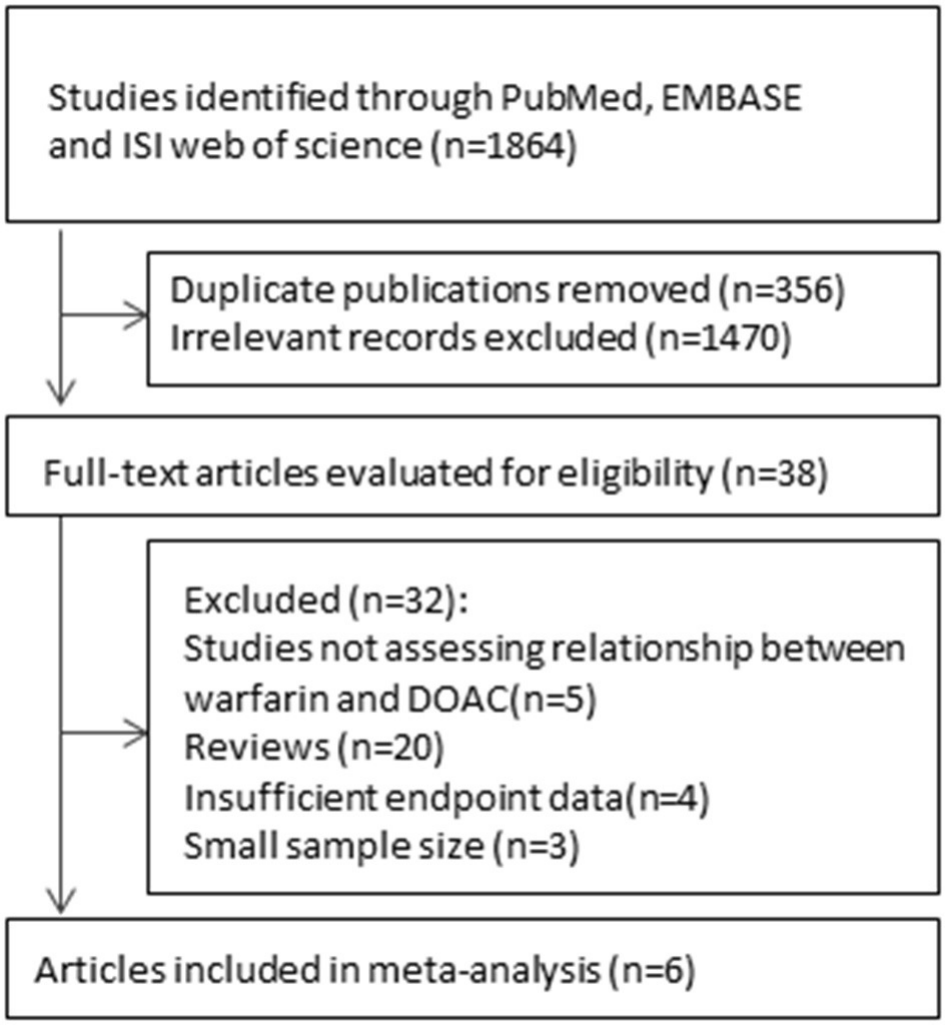
Flow chart for the study selection of randomized controlled trials.

As shown in the flow chart (Figure 1), the searched terms DOACs and VKAs showed 1,864 articles. After retrieving the titles and abstracts, 1,826 duplicate or irrelevant articles were excluded. A total of 38 articles were then reviewed in detail, and six controlled clinical trials with 22,040 participants were included in the current meta-analysis.

### Trials Included

A total of six published studies including four randomized controlled trials and two post-hoc analysis were included (6–11). The six studies both involved the subgroups according to age, sex, and renal function. Of the included six studies, two studies were subgroup analyses of HOKUSAI and RE-COVER. Although, in the included trials, not enrollment of all participants was randomized, no significant differences were observed concerning the baseline information. Table 1 summarized the major characteristics of the enrolled trials.

**Table 1:** Characteristics of the randomized controlled trials included in the meta-analysis.

**Drug class study year**	**Design**	**Study population*N***	**Age≤75 yrs*N* (%)**	**Sexmale*N* (%)**	**Creatinine clearance≥50 mL/min** ***N* (%)**
AMPLIFY(2013)	Randomized, Controlled Trial	5244	4495 (85.7)	3081 (58.8)	4456 (85.0)
EINSTEIN(2010)	Randomized, Controlled Trial	3449	3009 (87.2)	1960 (56.8)	3155 (91.5)
HOKUSAI(2013, 2018)	Randomized, Controlled Trial	8240	7136 (86.6)	4716 (57.2)	7699 (93.4)
RE-COVER and RE-COVER II(2014, 2017)	Randomized, Controlled Trial	5107	4578 (89.6)	3041 (59.5)	4814 (94.3)

### Efficacy Endpoints

**Figure 2:**
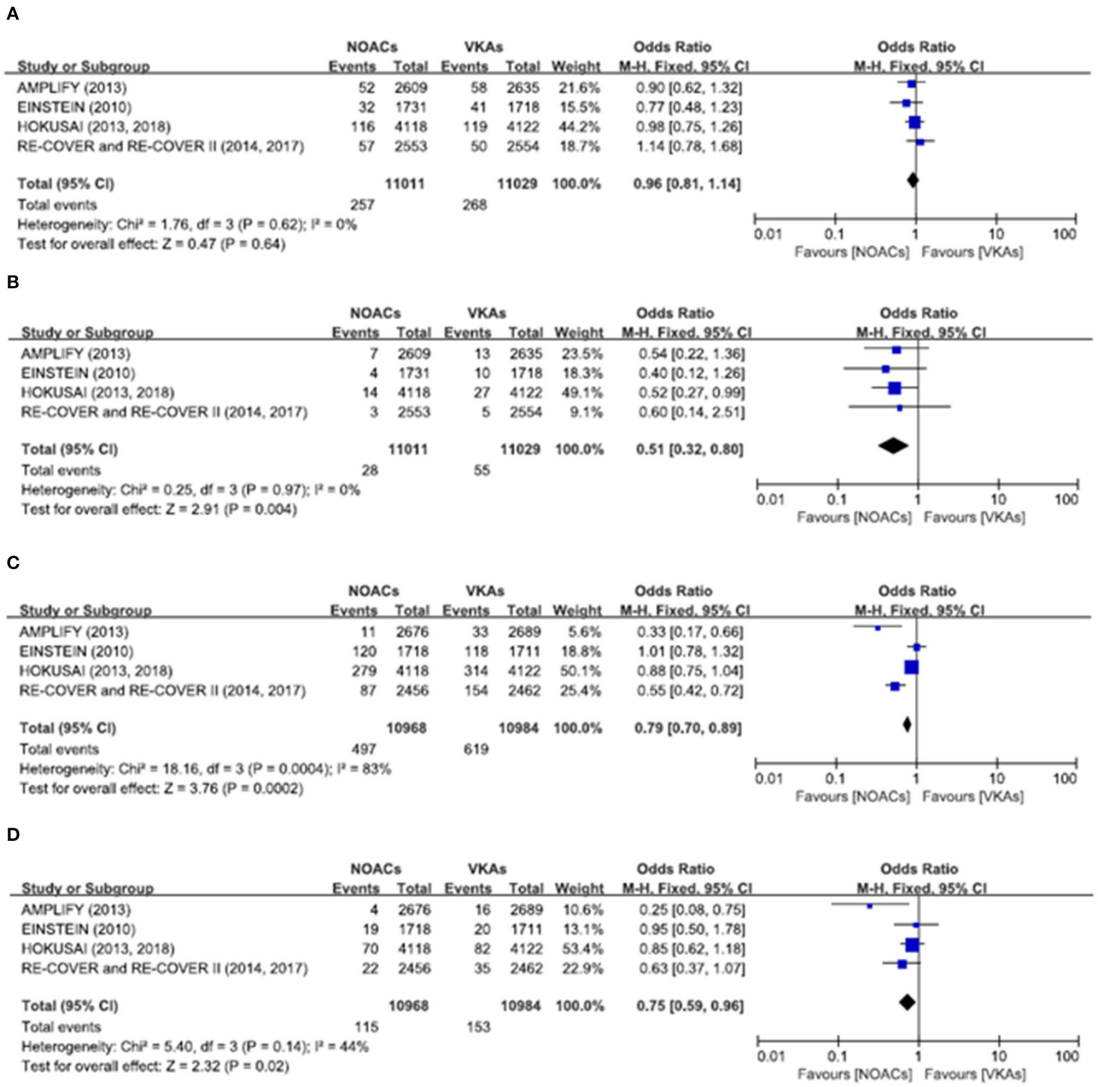
Efficacy and safety outcomes in our meta-analysis of the subgroups according to age. **(A)** Efficacy endpoint in patients ≤75 years; **(B)** efficacy endpoint in patients >75 years; **(C)** safety endpoint in patients ≤75 years; and **(D)** safety endpoint in patients >75 years.

**Figure 3:**
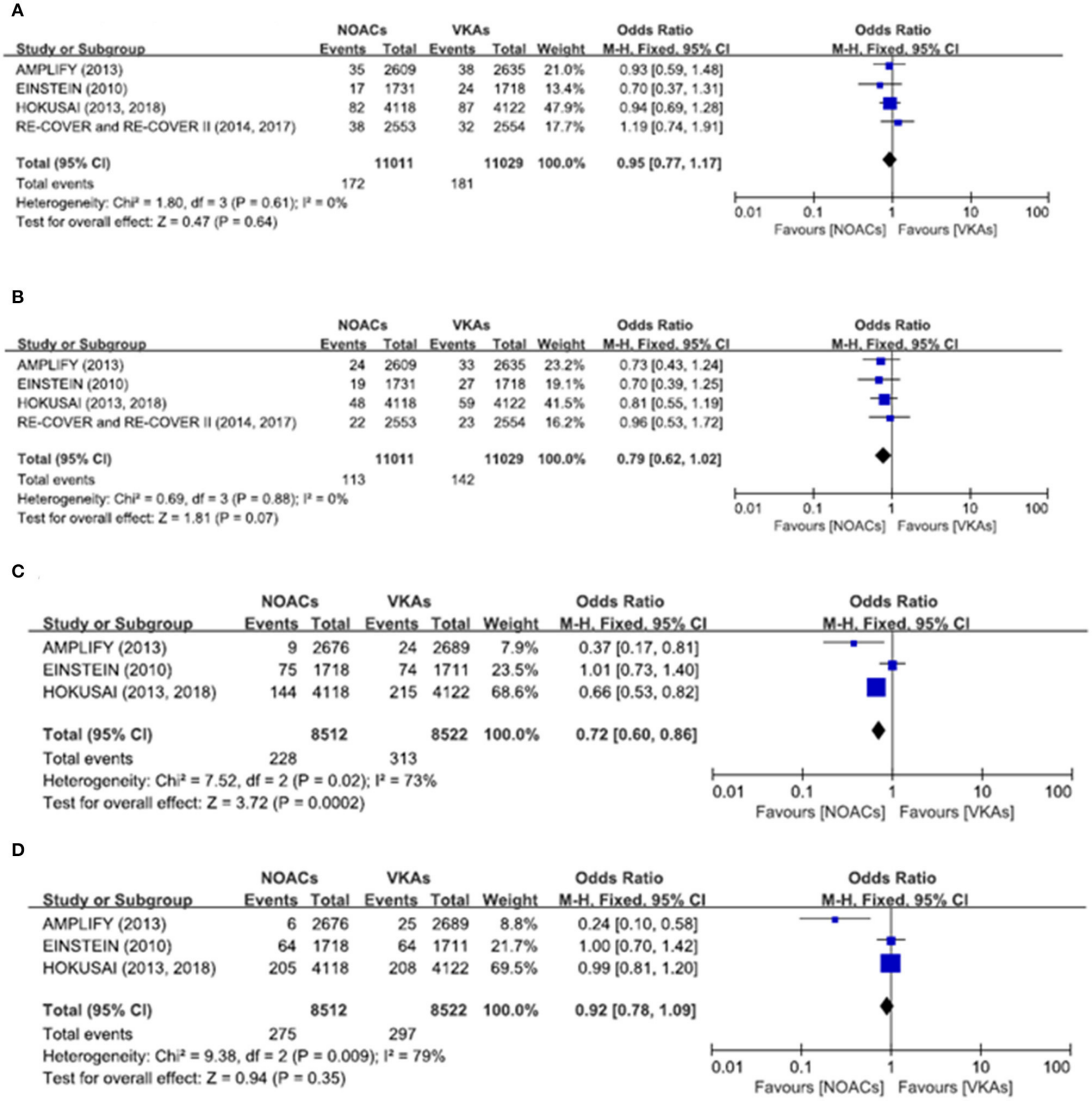
Efficacy and safety outcomes in our meta-analysis of the subgroups according to sex. **(A)** Efficacy endpoint in male patients; **(B)** efficacy endpoint in female patients; **(C)** safety endpoint in male patients; and **(D)** safety endpoint in female patients.

**Figure 4:**
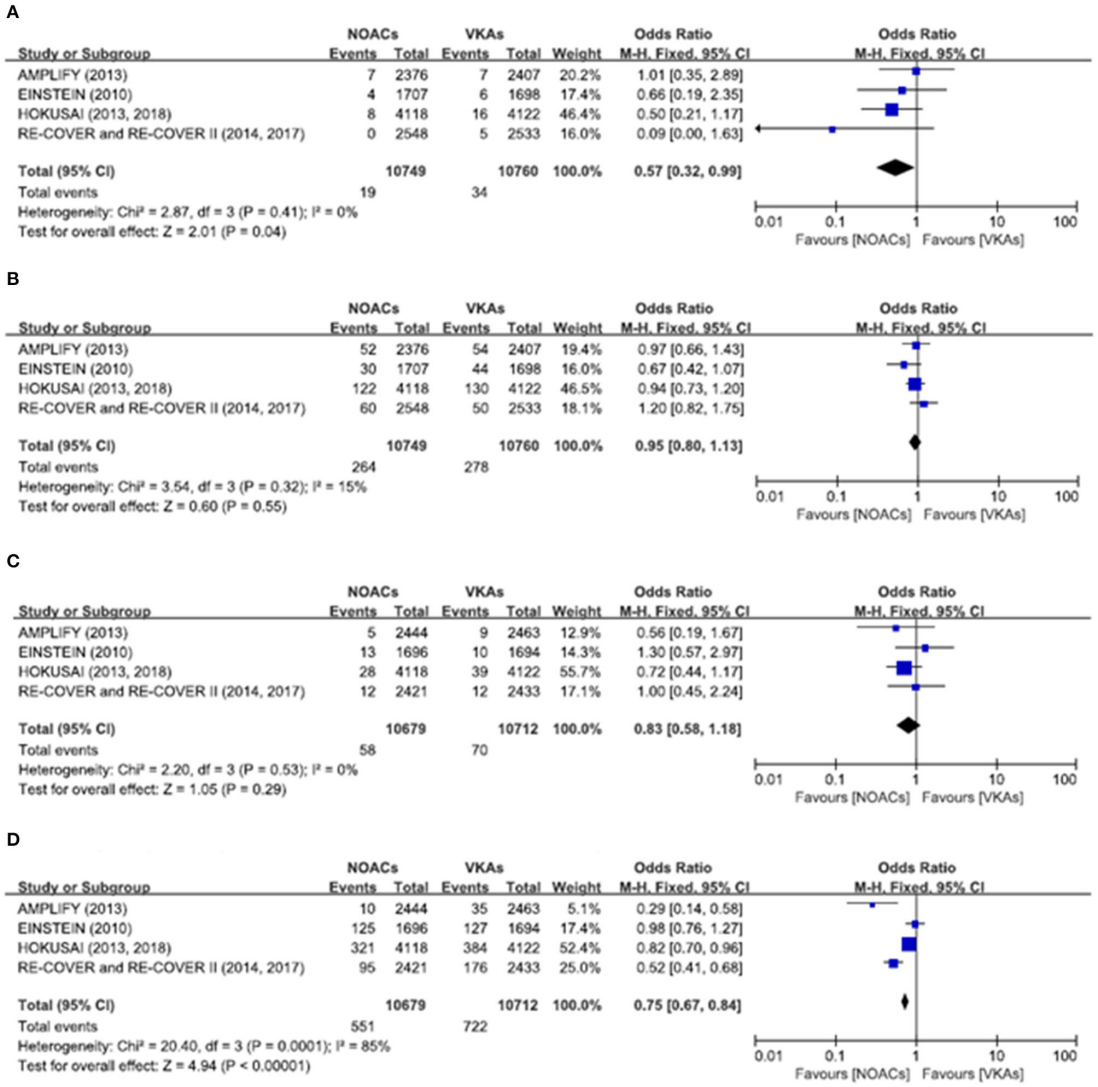
Efficacy and safety outcomes in our meta-analysis of the subgroups according to creatinine clearance. **(A)** Efficacy endpoint in patients with creatinine clearance <50 mL/min; **(B)** efficacy endpoint in patients with creatinine clearance ≥50 mL/min; **(C)** safety endpoint in patients with creatinine clearance <50 mL/min; and **(D)** safety endpoint in patients with creatinine clearance ≥50 mL/min.

There was no significant difference on the efficacy endpoints between DOACs and VKAs in terms of age ≤75 years (2.3 vs. 2.4%; OR: 0.96, 95% CI: 0.81–1.14; *P* = 0.64; *I*^2^ = 0%; Figure 2A), male (1.6 vs. 1.6%; OR: 0.95, 95% CI: 0.77–1.17; *P* = 0.64; *I*^2^ = 0%; Figure 3A), and creatinine clearance ≥50 ml/min (2.5 vs. 2.6%; OR: 0.95, 95% CI: 0.80–1.13; *P* = 0.55; *I*^2^ = 15%; Figure 4B), while DOACs were associated with a borderline higher efficacy of female (1.0 vs. 1.3%; OR: 0.79, 95% CI: 0.62–1.02; *P* = 0.07; *I*^2^ = 0%; Figure 3B), a significantly higher efficacy of age >75 years (0.3 vs. 0.5%; OR: 0.51, 95% CI: 0.32–0.80; *P* = 0.004; *I*^2^ = 0%; Figure 2B), and creatinine clearance <50 ml/min (0.2 vs. 0.3%; OR: 0.57, 95% CI: 0.32–0.99; *P* = 0.04; *I*^2^ = 0%; Figure 4A).

### Safety Endpoints

The primary safety bleeding endpoint was significantly reduced in DOACs as compared to VKAs on age ≤75 years (4.5 vs. 5.6%; OR: 0.79, 95% CI: 0.70–0.89; *P* = 0.0002; *I*^2^ = 83%; Figure 2C), age >75 years (1.0 vs. 1.4%; OR: 0.75, 95% CI: 0.59–0.96; *P* = 0.02; *I*^2^ = 44%; Figure 2D), male (2.7 vs. 3.7%; OR: 0.72, 95% CI: 0.60–0.86; *P* = 0.0002; *I*^2^ = 73%; Figure 3C), and creatinine clearance ≥50 ml/min (5.2 vs. 6.7%; OR: 0.75, 95% CI: 0.67–0.84; *P* < 0.00001; *I*^2^ = 85%; Figure 4D). There was no significant difference on safety endpoints between DOACs and VKAs in terms of female (3.2 vs. 3.5%; OR: 0.92, 95% CI: 0.78–1.09; *P* = 0.35; *I*^2^ = 79%; Figure 3D) and creatinine clearance < 50 ml/min (0.5 vs. 0.6%; OR: 0.83, 95% CI: 0.58–1.18; *P* = 0.29; *I*^2^ = 0%; Figure 4C).

## Discussion

Some clinical studies have shown that the use of DOACs may reduce recurrent VTE (12) and also reduce the high mortality rates (13) and long-term complications (14) of VTE. Besides, DOACs have the advantages of no need to monitor INR and reduced potential for drug interactions as compared to VKAs (15–18). Although clinical guidelines have first recommended the use of DOACs, there are limited data on safety and efficacy outcomes comparing DOACs with VKAs, especially assessing the impact of age, sex, and renal function on the safety and efficacy for the treatment of acute VTE.

In the RE-COVER trials, the patients receiving dabigatran displayed decreased incidence of VTE and VTE-related death but increased bleeding events in subgroups of increased age or decreased renal function (9). However, in our meta-analysis of the subgroups according to age, DOACs were associated with significantly higher efficacy in the age more than 75 years, but there was no significant difference on efficacy endpoints in the age <75 years. In addition, the primary safety bleeding endpoint was significantly reduced with DOACs as compared to VKAs both in the age <75 years and the age more than 75 years. Considering the higher bleeding incidents of the older patients or with decreased glomerular filtration rate (GFR), the pooled analysis might further validate which age and renal function subgroup would benefit most from DOAC as compared to VKA. Therefore, based on our meta-analysis results, the use of DOACs should be preferentially recommended in patients with age more than 75 years because of their advantage in preventing thrombosis and reducing bleeding events. In patients with age <75 years, DOACs and VKAs have similar efficacy, but DOACs are still superior to VKA in terms of bleeding.

In addition, in our meta-analysis of the subgroup according to sex, the primary safety endpoint was significantly superior in DOACs compared with VKAs in men, but there was no significant difference in efficacy endpoints. Interestingly, DOACs were associated with a borderline higher efficacy in women, suggesting probably differential drug sensitivity between men and women. As some women may present menstrual bleeding, under the protection of estrogen, or have other comorbidities, these factors could further affect the individual response. Thus, the in-depth mechanisms still warrant further and detailed subgroup analysis regarding different ages and sex.

Some clinical studies showed that lower GFRs was associated with higher bleeding risk but not with increased thromboembolic events, but the optimal anticoagulation treatment for patients with decreased renal function remains controversial (19–22). In our meta-analysis among patients with different renal functions, DOACs were associated with a significantly higher efficacy by preventing thrombosis in patients with GFR <50 ml/min, with no significant difference on safety endpoints. Interestingly, decreased bleeding risk in DOACs was noted in a subgroup with GFR more than 50 ml/min, with no significant difference on efficacy endpoints. Thus, in patients with creatinine clearance <50 ml/min, DOACs were associated with less recurrent VTE as compared with VKAs, indicating our preference to choose DOACs in patients with decreasing creatinine clearance.

The present meta-analysis also has several limitations. At first, the patients taking a reduced dose when creatinine clearance was <50, of other age groups, and women with the fertile age could not be in-depth investigated. Moreover, the follow-up periods were not similar in all the articles. It is of clinical significance to involve more high-quality RCTs in the future to solve the above shortcomings in the study.

## Conclusions

In conclusion, we performed a meta-analysis to assess the effect of age, sex, and renal function on the efficacy and safety of DOACs vs. VKAs for the treatment of acute VTE. DOACs have exhibited clinical preference among patients with acute VTE as compared to VKA with significantly decreased thrombosis events and lower bleeding complications, especially in patients with age more than 75 years and creatinine clearance <50 ml/min. Interestingly, DOACs also exhibited differential drug sensitivity between men and women, which warrants further mechanism study. This study provides more clinical evidence of DOAC applications in different sex, age, and renal function groups. Judging from the present meta-analysis, in patients with age more than 75 years and creatinine clearance <50 ml/min, DOACs are more strongly recommended in clinical practice.

## Data Availability Statement

The original contributions presented in the study are included in the article/supplementary material, further inquiries can be directed to the corresponding author/s.

## Author Contributions

JS, HW, and BL screened articles for the data inclusion, extraction, and quality assessment. BZ, JS, and CW participated in the design of the present meta-analysis. BZ, CW, and BL performed the statistical analysis and the revision. JS and BZ drafted the manuscript. All the authors have approved the final manuscript.

## Funding

This study was supported by the National Natural Science Foundation of China (81800390), the Key Research and Development Program of Shaanxi (2020KW-049 and 2021KWZ-25), and the Clinical Research Award of the First Affiliated Hospital of Xi'an Jiaotong University, China (No. XJTU1AF-CRF-2018-025).

## Conflict of Interest

The authors declare that the research was conducted in the absence of any commercial or financial relationships that could be construed as a potential conflict of interest.

## Publisher's Note

All claims expressed in this article are solely those of the authors and do not necessarily represent those of their affiliated organizations, or those of the publisher, the editors and the reviewers. Any product that may be evaluated in this article, or claim that may be made by its manufacturer, is not guaranteed or endorsed by the publisher.
